# Mechanisms of mercury (Hg) and methylmercury (CH_3_Hg^+^) removal by pristine and thiol-modified açaí waste nanobiochar

**DOI:** 10.1007/s11356-026-38003-4

**Published:** 2026-07-21

**Authors:** Matheus B. Soares, Luis C. C. Hurtarte, Tom Sizmur, Susan Nehzati, Luís R. F. Alleoni

**Affiliations:** 1 Department of Soil Science, Luiz de Queiroz College of Agriculture (ESALQ), University of São Paulo (USP), Piracicaba, 13418900 Brazil; 2 Institute of Earth Surface Dynamics, University of Lausanne, Lausanne, 1015 Switzerland; 3 Diamond Light Source, Harwell Science and Innovation Campus, Didcot, OX11 0DE UK; 4 Department of Geography and Environmental Science, University of Reading, Reading, RG6 6UR UK

**Keywords:** Thiol-functionalization, Biochar, Circular economy, Environmental remediation, Spectroscopy, EXAFS

## Abstract

Mercury (Hg) contamination remains a global environmental challenge, driven by sources such as artisanal gold mining, industrial emissions, and coal combustion. Developing efficient and sustainable remediation strategies is therefore critical. Here we evaluate for the first time the mechanisms controlling Hg and methylmercury (CH_3_Hg^+^) removal using nanobiochar derived from açaí (*Euterpe oleracea* Mart) seed residues and its thiol-functionalized counterpart. Nanobiochar (< 50 nm) was produced via pyrolysis at 600 °C and chemically modified to enhance sorption performance. Thiol functionalization increased maximum sorption capacities by 29% for Hg^2+^ (from 299 to 385 mg g^−1^) and 61% for CH₃Hg⁺ (from 102 to 164 ng g^−1^), while also reducing desorption. Spectroscopic analyses (XPS and EXAFS) revealed that Hg immobilization was primarily driven by the formation of stable Hg–S species, including metacinnabar (β-HgS), highlighting a shift from weaker ionic interactions to covalent bonding. This mechanistic evidence demonstrates that surface functionalization not only enhances sorption capacity but also promotes long-term stability of retained Hg species. By coupling waste valorization with advanced spectroscopic insights, this study provides a novel framework for designing high-performance materials for Hg remediation in contaminated environments.

**Supplementary Information:**

The online version contains supplementary material available at 10.1007/s11356-026-38003-4.

## Introduction

Mercury (Hg) contamination poses several environmental and health risks due to its persistent and toxic nature (Gworek et al. 2020). Mercury can enter the environment through natural processes such as volcanic activity, yet the major contributors to increased soil and water contamination are human activities such as coal burning, mining, and improper disposal of Hg-containing products (Natasha et al. 2020). Once in the environment, Hg can transform into methylmercury (CH_3_Hg^+^), a highly toxic compound that biomagnifies up food webs and bioaccumulates in fish and shellfish, posing serious health risks (Wu et al. 2024). Chronic exposure to Hg can lead to neurological and developmental deficits, especially in fetuses and young children, and is associated with cardiovascular disease in adults (Kang et al. 2024). In addition, Hg contamination can disrupt ecosystems by affecting the reproduction and survival of several species, and thus threatening biodiversity (Driscoll et al. 2013; Bello et al. 2023; Soares et al. 2026).

The World Health Organization estimates that tens of millions of people are at risk of exposure to Hg, which can lead to serious health consequences, especially in developing countries, where regulations and protective measures are generally weaker (WHO 2008). Faced with this problem, several strategies have been used to remediate Hg contamination, each focused on different contexts. Researchers have sought to develop different materials and techniques with the potential to remove or immobilize Hg species in soils and water (Janeiro-Tato et al. 2021; Padhye et al. 2023; González-Reguero et al. 2023). Techniques such as phytoremediation, which uses plants to take up Hg, and bioremediation, employing microorganisms to detoxify Hg, have shown promise (Tanwer et al. 2022; Bhat et al. 2022). In industry, activated carbon is widely used to capture elemental mercury (Hg⁰) released by the chimneys of coal-fired power plants (Sjostrom et al. 2010), and activated carbon and biochar are being developed to immobilize Hg in contaminated areas, expanding the options for dealing with this type of pollution (Tran et al. 2024).

Several techniques have been developed to modify and enhance the sorption potential of organic materials. However, limitations such as low contaminant selectivity, slow retention kinetics, and limited sorption capacity remain challenges (Liu et al. 2022). To overcome these issues, researchers have explored nanotechnology and chemical surface modification of pyrolyzed biomaterials. The synthesis of biochar nanoparticles typically involves mechanical processes such as ball milling or ultrasonication, which reduce biochar derived from biomass pyrolysis into nanometer-sized particles (Naghdi et al. 2017). This transformation into nanobiochar increases surface area and reactivity, and improves its efficiency in environmental remediation (Ramanayaka et al. 2020). On the other hand, chemical modifications, such as oxidation, sulfonation, or the incorporation of metal oxides, further enhance the material’s sorption potential and make it highly effective at retaining Hg species (Tan et al. 2016; Sun et al. 2023; Zhao et al. 2024).

Selenium (Se)-containing biomaterials have been investigated for Hg remediation due to their ability to form stable Hg–Se species (Jing et al. 2024). However, their application is often constrained by the potential toxicity of Se, its narrower environmental safety thresholds, and the risk of secondary contamination due to Se mobility (Qu et al. 2023). In contrast, sulfur (S)-based modifications are generally more environmentally compatible, widely available, and capable of forming highly stable Hg–S bonds with strong affinity and persistence under a range of environmental conditions (Park et al. 2019). These modifications strengthen binding interactions and increase sorption efficiency, addressing challenges in contaminant immobilization. In this context, nanobiochar presents a sustainable solution for waste management and water purification.

The Brazilian açaí (*Euterpe oleracea*, Mart) industry, concentrated in the northern states, generates around 1.7 million tons of fruit annually (Brazilian Institute of Geography and Statistics 2023), with seeds comprising 80% of their weight (Polidori et al. 2024). This results in over 1 million tons of seed residue each year, and its improper disposal contributes to pollution, greenhouse gas emissions, and waste accumulation (Melo et al. 2021). Converting these residues into nanobiochar mitigates waste disposal issues and produces a highly functional material for water filtration. In the Amazon, nanobiochar can be employed to treat Hg-contaminated water near artisanal gold mining sites, thus reducing ecological damage and health risks from Hg bioaccumulation in the food chain (Sato et al. 2020). The high adsorption capacity of biochar also makes it suitable for industrial applications, such as filtration systems in power plants and wastewater treatment facilities worldwide (Ma et al. 2022; Xiao et al. 2023).

Sorption/desorption isotherms have been widely used to model Hg retention because they provide indirect evidence of Hg–sorbent interactions (Yang et al. 2008; Liao et al. 2009; Moharem et al. 2019; Gao et al. 2023). However, precise mechanistic understanding requires advanced spectroscopic techniques such as Extended X-ray Absorption Fine Structure (EXAFS) and X-ray Photoelectron Spectroscopy (XPS). EXAFS enables detailed characterization of the local atomic environment of Hg, distinguishing between surface complexation, precipitation, and structural incorporation (Hesterberg et al. 2001), while XPS provides surface-sensitive information on Hg oxidation states and bonding environments (Behra et al. 2001). Here, sorption/desorption experiments are directly combined with synchrotron-based EXAFS and surface-sensitive XPS to establish explicit links between macroscopic retention behavior and molecular-scale binding mechanisms, allowing a more robust evaluation of nanobiochar stability and long-term performance under environmentally relevant conditions.

In this study, we evaluated the potential and mechanisms of thiol-modified nanobiochar for immobilizing Hg^2+^ and CH_3_Hg^+^ through combined sorption and desorption experiments. This approach couples conventional batch experiments with advanced spectroscopic analyses to simultaneously resolve Hg speciation and binding configurations. We employed wet chemical extractions, XPS for surface chemical characterization, and EXAFS for detailed structural analysis of Hg species. This integrated methodology allowed us to elucidate the molecular mechanisms governing Hg retention and to demonstrate that thiol functionalization enhances Hg immobilization through the formation of strong, stable Hg–S bonds, thereby reducing Hg mobility and bioavailability in contaminated systems.

## Material and methods

### Reagents

All reagents used in this study were of analytical grade or higher purity. Ethanol (purity ≥ 99.8%) was purchased from Synth (Diadema, Brazil). The silane (3-mercaptopropyl)trimethoxysilane (3-MPTS, purity ≥ 95%) coupling agent was obtained from Merck (Darmstadt, Germany) and used for the thiol functionalization of the nanobiochar.

Mercury stock solutions were prepared using mercury chloride (HgCl_2_, purity ≥ 99%, Merck, Darmstadt, Germany) and a standard methylmercury solution (CH_3_Hg^+^, Merck, Darmstadt, Germany). Hydrochloric acid (HCl, 37% w/w, Merck, Darmstadt, Germany) and sodium hydroxide (NaOH, purity ≥ 98%, Merck, Darmstadt, Germany) were used to adjust the pH during the sorption experiments.

Sodium nitrate (NaNO_3_, purity ≥ 99%, Merck, Darmstadt, Germany) was used as the supporting electrolyte for zeta potential measurements. Sodium chloride (NaCl, purity ≥ 99.5%, Merck, Darmstadt, Germany) was used as the supporting electrolyte in sorption experiments, while calcium chloride (CaCl_2_, purity ≥ 99%, Merck, Darmstadt, Germany) was used as the desorption solution during desorption tests. Ultrapure deionized water (≥ 18.2 MΩ cm^−1^) was used for the preparation of all solutions, suspensions, washing procedures, and analytical determinations.

### Production and chemical modification of nanobiochar

Nanobiochar was produced from açaí seed residues, taking advantage of an abundant by-product from the agroindustry. This approach provides locally available and cost-effective feedstock, particularly in regions affected by Hg contamination, where it can support remediation strategies while minimizing transportation requirements.

To produce the biochar, the feedstock was placed in a sealed reactor to exclude oxygen, and the reactor’s furnace was heated at a rate of 5 °C min^−1^ until reaching a final pyrolysis temperature of 600 °C. This temperature was chosen based on previous research that demonstrated the effectiveness of biochars pyrolyzed at around 600 °C for removing Hg^0^ and Hg^2+^ species (Li et al. 2015; Shen et al. 2015; Yang et al. 2016; Park et al. 2019). A ball mill was used to convert the biochar into nanobiochar (< 50 nm) due to its low cost, efficiency, and environmentally friendly nature (Pratap et al. 2021). To prevent nanoparticle interaggregation, the pristine nanobiochar was dispersed in ethanol (1:20) and sonicated at 50 kHz for 30 min using a probe-type ultrasonic vibrator (Dong et al. 2018).

Thiol-modified nanobiochar was prepared by mixing a solution of water, ethanol (≥ 99.8% purity, Synth, Diadema, Brazil), and (3-Mercaptopropyl)trimethoxysilane (3-MPTS, ≥ 95% purity, Merck, Darmstadt, Germany) (3.6 mL of water, 114 mL of ethanol, and 2.4 mL of 3–MPTS). Three grams of nanobiochar were placed in a 500 mL agate crucible, and 120 mL of the 3-MPTS solution were added dropwise with a Pasteur pipette. The mixture was homogenized with an agate ball mill, without applying force, only to mix the material. After milling, the thiol-modified nanobiochar was washed three times with ethanol and deionized water, and then freeze-dried for 48 h (Lyu et al. 2020).

### In situ high-energy X-ray diffraction (XRD) measurements during pyrolysis

Temperature-resolved high-energy XRD patterns were collected at the PAINEIRA beamline of the National Synchrotron Light Laboratory (Sirius) at the Brazilian Center for Research in Energy and Materials (CNPEM), Brazil, to observe the evolution of inorganic minerals in the açaí residue during pyrolysis, which may precipitate Hg and CH_3_Hg^+^ if retained in the ash portion of the nanobiochar. The X-ray beam wavelength was 0.24 Å.

For the in situ experiment, the açaí sample was previously dried, ground, and pressed in quartz capillary tubes. Subsequently, the sample and the sample holder were placed in an Oxford FMB furnace perpendicular to the X-ray beam. The sample was heated in hot air from 22 to 600 °C at a rate of 5 °C min^−1^ under an inert gas atmosphere (N_2_ – gas flow of 2 mL min^−1^). During pyrolysis, a PiMega 450D X-ray detector was used to collect the XRD patterns at a rate of 1 scan every 5 min. The diffraction patterns were identified using the International Centre for Diffraction Data (ICDD) standards (ICDD 2024).

### Physicochemical characterization of nanobiochar

The total contents of carbon (C), nitrogen (N) of the biochar were obtained via combustion and quantified in a CHN elemental analyzer (Carmo and Silva 2012). Subsequently, the C:N ratios were calculated. The specific surface area (SSA) was determined by the N_2_-BET method, utilizing nitrogen sorption and BET isotherm fitting (Song and Guo 2012). The morphology and particle size of the nanobiochar surface were examined with a transmission electron microscope (TEM; FEI TECNAI G^2^ F20 HRTEM, Fei Company). For this analysis, nanobiochars were suspended in deionized water (0.1 g L^−1^ concentration), pipetted onto a copper grid with carbon film, air-dried, and imaged at 200 kV. TEM imaging captured the samples at magnifications of 500 and 200 nm.

The surface charge of the nanobiochars was evaluated using zeta (ζ) potential measurements. Samples were diluted in 0.01 M NaNO_3_ (≥ 99% purity, Merck, Darmstadt, Germany), achieving a final pH of 7.8 ± 0.1 and a concentration of 0.1 g L^−1^. These samples were loaded into capillary cells and analyzed with a Malvern Zetasizer Nano Z (Malvern PANalytical), with quality assurance provided by an external Malvern standard exhibiting a potential of −42 ± 4.2 mV.

Proximate analysis was performed to estimate the solid composition of the biochar. The contents of fixed carbon (C-fixed), volatile carbon (C-volatile), and ash were determined by measuring the mass loss after heating in a muffle furnace at different temperatures following ASTM standards (ASTM 2007). The nanobiochar data were verified for normality (Shapiro-Wilk test) and due to the non-normality of the data, the means were subsequently compared using the 95% confidence intervals created by bootstrapping.

### Sorption isotherm

The potential retentions of Hg^2+^ and CH_3_Hg^+^ on pristine and thiol-modified nanobiochar were evaluated through batch sorption experiments (*n* = 6). Sorption isotherms were performed individually for each Hg species to assess their independent potential for retention on the nanobiochars, rather than examining competition for sorption sites. Half of the replicates (*n* = 3) were used for spectroscopic analysis, while the remaining replicates (*n* = 3) were reserved for subsequent desorption studies.

Sorption experiments were conducted to estimate the maximum retention capacity of Hg^2+^ and CH_3_Hg^+^on pristine and thiol-modified nanobiochar at a pH close to the natural pH of the nanobiochar (pH = 7.8). The pH was adjusted using either 0.1 mol L^−1^ HCl (37% w/w, Merck, Darmstadt, Germany) or 0.1 mol L^−1^ NaOH (≥ 98% purity, Merck, Darmstadt, Germany), and monitored throughout the experiment. Stock solutions of 10 mg L^−1^ Hg^2+^ and 1 mg L^−1^ CH_3_Hg^+^ were prepared using reagent-grade HgCl_2_ (≥ 99% purity, Merck, Darmstadt, Germany) and CH_3_Hg^+^ (Merck, Darmstadt, Germany) in ultrapure water. These stock solutions were diluted to final concentrations of 6000 μg L^−1^ Hg and 200 μg L^−1^ CH_3_Hg^+^, which are levels representative of Hg concentrations in wastewater.

For the sorption assays, Hg concentrations of 0, 10, 20, 30, 40, 50, 100, 150, 300, 450, and 600 μg L^−1^ were tested, while concentrations of 0, 3, 7, 15, 30, 45, 60, 90, 120, 150, and 200 ng L^−1^ were used for CH_3_Hg^+^. These concentration ranges allowed the evaluation of the sorption behavior and maximum retention capacity for both pristine and thiol-modified nanobiochar.

Samples of pristine and thiol-modified nanobiochar (1 g L^−1^) were equilibrated with a 0.15 mol L^−1^ NaCl electrolyte solution for 24 h at 25 °C using a rotary shaker. Supernatant solutions were separated via centrifugation at 10,000 rpm for 10 min and filtered through a < 0.1 μm Durapore® membrane filter. The concentration of Hg sorbed on the nanobiochars was calculated as the difference between the initial and equilibrium solution concentrations. The sorption data were fitted to a Langmuir isotherm using an Excel-based nonlinear solver. Model selection criteria included the Akaike Information Criterion, model efficiency, and the sum of squared errors.

All the samples with Hg and CH_3_Hg^+^ were previously distilled before direct ethylation to reduce potential interferences, and then quantified via ICP-MS (Muller et al. 2019). The concentration was measured using a Brooks Rand MERX Hg Speciation GC and Pyrolysis coupled to an ELAN DRC-e ICP-MS.

### Desorption process of Hg^2+^ and CH_3_Hg^+^

Desorption was performed on the pristine and thiol-modified nanobiochar samples after sorption of Hg^2+^ and CH_3_Hg^+^, which were recovered after filtration, washing, and lyophilization. Briefly, desorption solutions containing 0.01 mol L^−1^ CaCl_2_ (≥ 99% purity, Merck, Darmstadt, Germany) at pH = 7.8 were used to desorb Hg from the nanobiochars. Desorption was conducted for 24 h with initial intervals of 5 min, and subsequently of 1, 2, and 3 h. The amount of desorbed Hg was calculated as the difference between the amounts of sorbed and desorbed Hg.

The Hg and CH_3_Hg^+^ contents in the solution during the desorption process were determined in the same way as the Hg and CH_3_Hg^+^ contents of the sorption study, using Brooks Rand MERX Hg Speciation GC and Pyrolysis coupled to an ELAN DRC-e ICP-MS.

The desorbed Hg concentrations were modeled using pseudo-first and pseudo-second-order kinetic models to evaluate the Hg desorption behavior from the nanobiochars and the determination of the kinetic constants and the desorption capacity at equilibrium (*Qₑ*). The pseudo-first-order model was initially fitted to the experimental data, assuming that the desorption could be limited by a simple desorption process, while the pseudo-second-order model was used to evaluate whether the desorption process was more complex because it involves chemical interactions. The models were fitted by minimizing quadratic errors, and the kinetic parameters were calculated based on the model equations to provide a detailed understanding of the Hg desorption dynamics in the studied materials.

### Speciation of carbon, oxygen, sulfur, and mercury on the surface of the nanobiochar

The speciation of carbon, oxygen, sulfur, and mercury on the surface of pristine and thiol-modified nanobiochar before and after Hg and CH_3_Hg^+^ sorption was analyzed by X-ray photoelectron spectroscopy (XPS). The analysis was performed on powder samples previously dispersed on double-sided carbon tape. For this purpose, an XPS/UVS spectrometer (Thermo Scientific) with an Al anode monochromator, at 10–14 kV energy and < 1 eV resolution was used. The position of the binding energy peaks was calibrated by setting the C 1 s peak to 284.8 eV for all pristine and thiol-modified nanobiochar samples (Martinson and Reddy 2009). Baseline fitting was performed using the Shirley Eq. (1), and the relative atomic content was calculated from the integration of the C 1 s, S 2p, O 1 s, and Hg 4f spectral lines.

1 $$B\left(E\right)=Y_2+\left(Y_1-Y_2\right)\;\frac{{\displaystyle\int\limits_E^{E1}}\left[y\left(E'\right)-B\left(E'\right)\right]\;dE'}{\int\limits_{E2}^{E1}\left[y\left(E'\right)-B\left(E'\right)\right]\;dE'}$$

where *B(E)* is the background intensity at energy (*E*), *y*(*E´*) is the measured raw signal intensity at energy (*E´*), *E*_1_ is the start (low binding energy/high kinetic energy) of the peak region where the background is lower, *E*_2_ is the end (high binding energy/low kinetic energy) of the peak region where the background is higher, and *Y*_1_ and *Y*_2_ are the measured intensities at the endpoints *E*_1_ and *E*_2_, respectively.

The spectra were fitted using Gaussian–Lorentzian functions by Voigt function (Eqs. 2–5), and the high-resolution spectra of S 2p and Hg 4f were analyzed taking into account the doublet of S 2p (1/2 and 3/2) and Hg 4f (5/2 and 7/2) due to spin–orbit splitting, with a binding energy (BE) difference of 0.6 ± 0.2 eV, equal full width at half maximum (FWHM) and an approximate area ratio between 3:2.

2 $$V\left(x',y\right)=\frac y\pi\int\limits_{-\infty}^\infty\frac{e^{-t^2}}{\left(x'-t\right)^2+y^2}dt$$

where

3 $$x'=2\sqrt{{log}_e2\frac{lhm}{ghm}}$$

and

4 $$y=2\sqrt{{log}_e2\frac{lhm}{ghm}}$$

where *ghm* and *lhm* are defined as the full width at half maximum of the Gaussian and Lorentzian functions, respectively. Finally, a mixing parameter, *M*, with a value between 0.0 and 1.0, with 0.0 being a pure Gaussian and 1.0 a pure Lorentzian, was defined as:

5 $$M=\frac{lhm}{lhm+ghm'}$$

where *M* is a numerical representation of the mixing that is done in Eq. 2 (the amounts of Gaussian and Lorentzian character in the Voigt function are controlled by the widths of its Gaussian and Lorentzian components).

### Structural characterization of Hg species

Extended X-ray absorption fine structure (EXAFS) spectroscopy of Hg was conducted at beamline I18 of the Diamond Light Source, UK. Analyses were performed on pristine and thiol-modified nanobiochar samples after the sorption of HgCl_2_ and CH_3_Hg^+^. Solid samples were prepared as thin films on polyimide tapes (Kapton™) fixed to the beamline sample holder.

Measurements focused on the L_3_ edge of Hg (12,284 eV) using a Si(111) double crystal monochromator and were performed in fluorescence mode with a four-channel Vortex detector. The beam was adjusted to a size of approximately 100 µm × 100 µm, ensuring high accuracy in localized analyses (Mosselmans et al. 2009). The scans covered the pre-edge region (100 eV below the edge), the absorption edge (50–100 eV around the edge), and the EXAFS region (~ 1000 eV above the edge, equivalent to ~ 12 Å^−1^ in *k*-space) (Nehzati et al. 2022). Multiple scans were performed on each sample (*n* = 3) to improve the signal-to-noise ratio. Energy calibration was performed using Au foil, while beam stability was monitored during all measurements.

Background subtraction was performed using the Larch software, which began with the normalization of the incident and transmitted beam intensities. The reference energy was set using the first inflection point of the absorption edge of an Au foil (11,919 eV). Any shift in the energy axis was determined and applied according to the collected measurements, ensuring proper alignment with the reference standard.

Background subtraction was performed using the Larch software, beginning with the normalization of the incident and transmitted beam intensities. The reference energy was defined as the first inflection point of the Au L_3_-edge, set at 11,919 eV. All spectra, including experimental samples and Hg reference standards, were calibrated using the same Au foil collected during the beamtime session. Minor energy shifts (≤ ± 0.3 eV) were applied when necessary to correct for small alignment discrepancies, ensuring consistency across all datasets during the Linear Combination Fitting (LCF).

LCF analysis was performed in *k*-space to quantify the relative contributions of reference species to the EXAFS spectra of the samples. The spectra were processed by background removal to isolate the EXAFS oscillations and transformed into *k*-space with a Kaiser-Bessel window in a range of 1 to 8 Å^−1^. The fitting was conducted as a linear combination of reference spectra, selected based on different structural environments of the absorber and considering only standards with a minimum contribution of 10% to the spectral reconstruction. The references used were HgCl_2_, Hg bound to cysteine ​​(Hg-cysteine), HgSO_4_, HgS–β, Hg–Se, CH_3_Hg^+^, and CH_3_Hg^+^ bound to hydroxyl (CH_3_Hg–OH). The minimization of the statistical error was performed using the *χ*^2^ and *R*-factor reduction criteria.

## Results and discussion

### Structural and morphological characteristics of nanobiochar

**Fig. 1:**
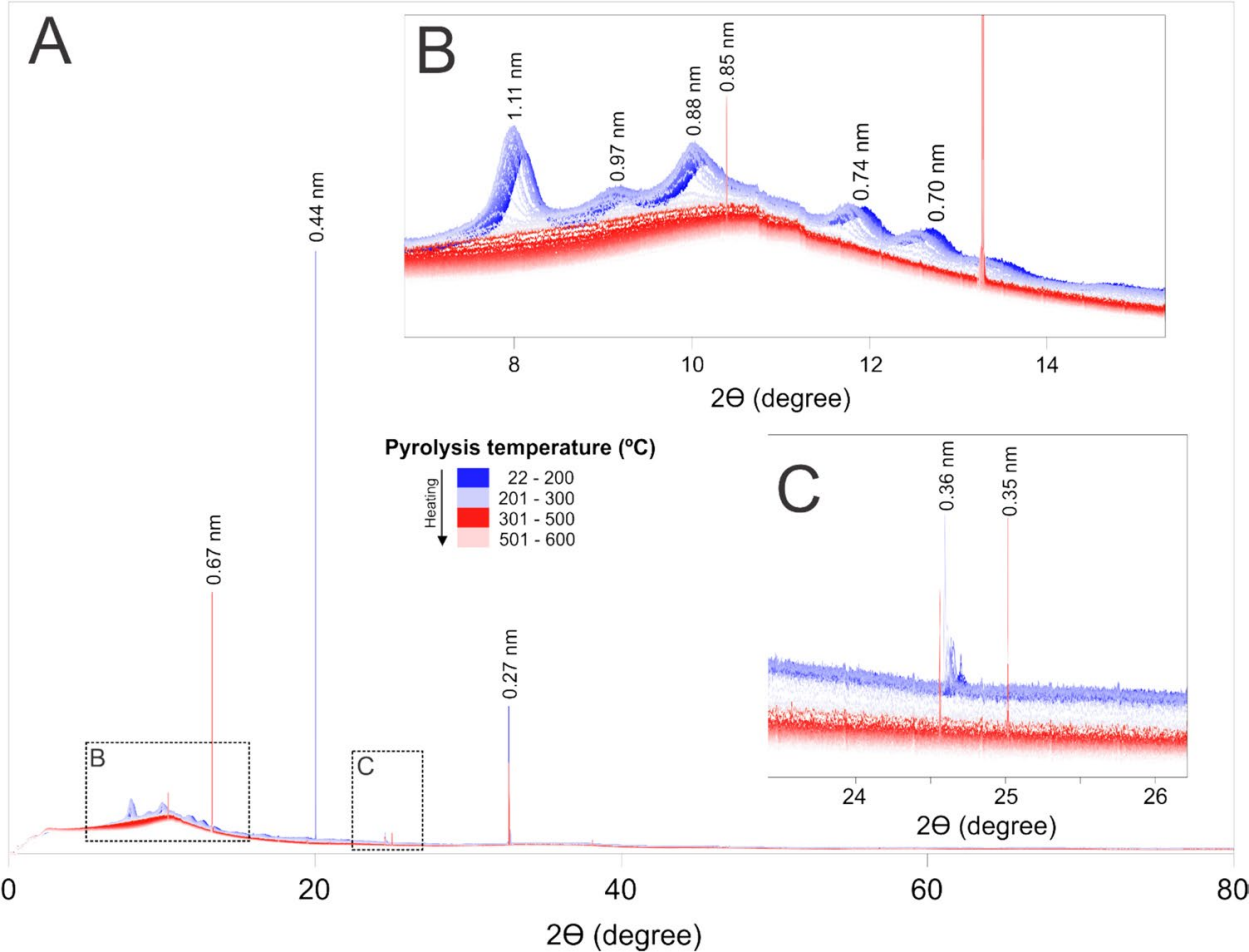
In situ synchrotron XRD (λ = 0.459 Å) of açaí seeds during pyrolysis (22–600 °C; 5 °C min.^−1^). Blue to Red indicates temperature increasing. **A** Full XRD pattern. **B** Low-angle region (interlayer spacing). **C** High-angle region (short-range order)

X-ray diffraction patterns obtained in situ during pyrolysis showed the devolution of the crystal structure of açaí seeds as temperature increased (Fig. 1). As the pyrolysis temperature increased, the crystal structure was altered in a way that promoted a decrease in the intensity and number of peaks. The peaks at 1.11 (long-chain fatty acid), 0.97 (fatty acid), and 0.88 (surfactants/quaternary ammonium) nm became progressively less intense or disappeared at higher temperatures. This suggests the disorganization or decomposition of structures associated with temperature-sensitive organic compounds (Dini et al. 2024).

Similarly, peaks at 0.44 (aromatic compounds) and 0.36 (carbohydrate) nm disappeared with increasing temperature above 300 °C, indicating thermal degradation and carbonization of the organic structures of these compounds (Kaur et al. 2024). Carbohydrates, such as cellulose, have a low thermal stability at temperatures above 280ºC, resulting in the breaking of polymer chains and the release of volatile compounds such as gases, levoglucosan sugars, and carbonaceous residues (Van de Velden et al. 2010). In contrast, peaks at 0.67 nm (Graphene/CaCO_3_) emerged at higher temperatures, suggesting the persistence or neoformation of crystalline mineral phases.

Some reflections, such as at 0.37 (Graphene/CaCO_3_), 0.35 [Al_2_O_3_/SiO_2_/Ca_5_(PO_4_)_3_], and 0.27 nm (Graphene/Fe_3_O_4_) remained even at high temperatures, indicating the presence of thermally stable inorganic components or the transformation of organic matter into resistant carbonized structures. This transition is consistent with the evolution from amorphous to more ordered carbon structures during pyrolysis (Nguyen et al. 2010), supported by peak broadening in the 0–20° 2ϴ range. This was evidenced by the broadening of the peaks (0–20º 2ϴ) and the reduction in their intensity.

**Fig. 2:**
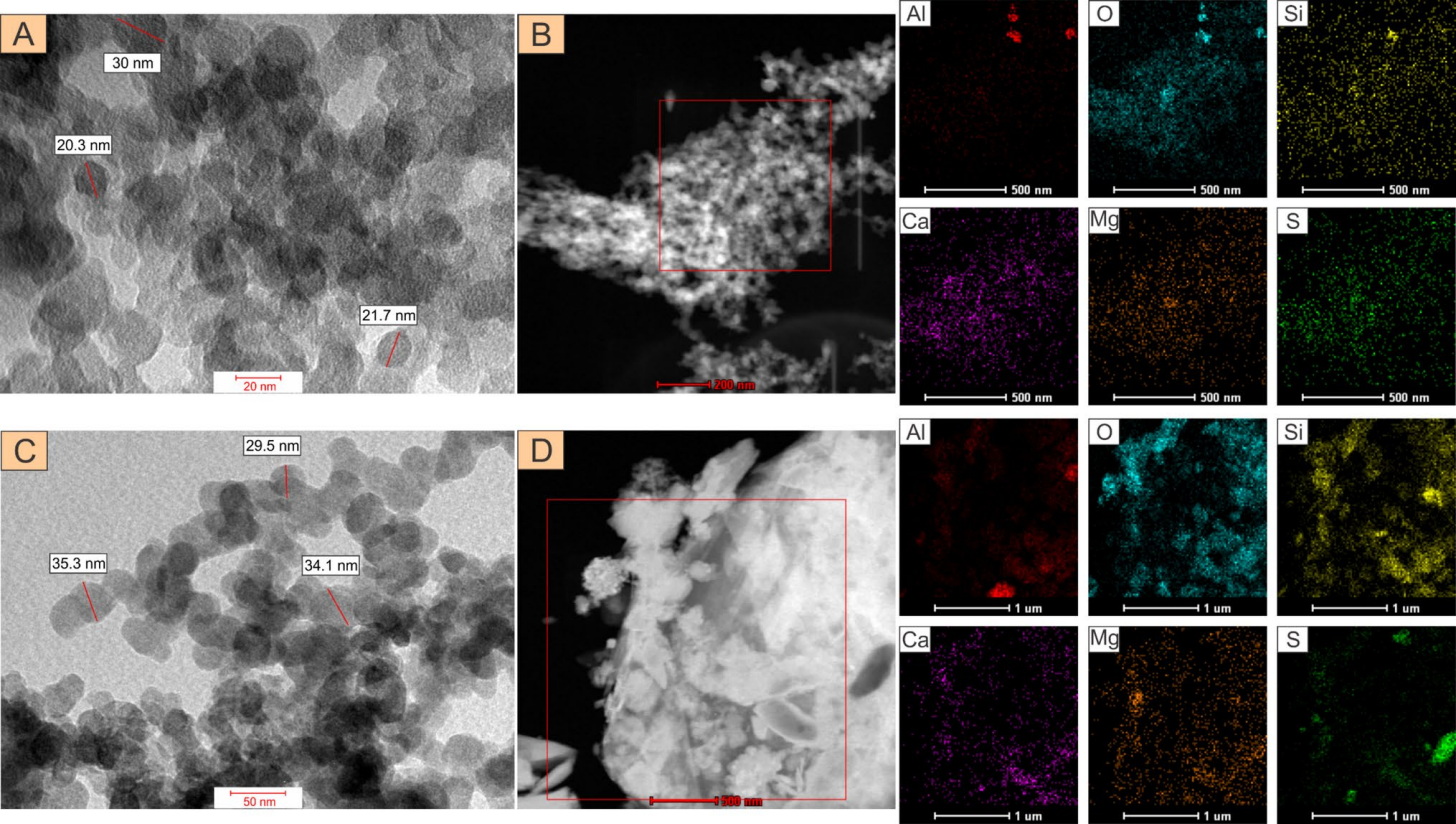
Transmission Electron Microscopy images of pristine (**A** and **B**) and thiol-functionalized nanobiochar (**C** and **D**). The red squares in panels **B** and **D** indicate regions selected for elemental mapping for Al, O, Si, Ca, Mg, and S. The images were captured at resolutions of 20 nm, 200 nm, 500 nm, and 1 μm, with an accelerating voltage of 200 kV

The formation of nanobiochar particles was confirmed by TEM images (Fig. 2), which showed uniform, rounded particles with well-defined morphology. The milling process produced particles with an average size of approximately 30 nm. However, aggregation among nanoparticles was observed, likely due to their high surface energy (Oleszczuk et al. 2016).

Aggregation is expected under natural conditions, such as in soils, and can significantly influence nanobiochar behavior. While aggregation reduces the available surface area and reactive sites, potentially limiting reactivity and contaminant interaction, it also decreases mobility and environmental risk by restricting dispersion and bioavailability. Thus, aggregation represents a trade-off between reactivity and environmental safety (Xing et al. 2023).

### Physicochemical and surface chemical changes after thiol modification

**Fig. 3:**
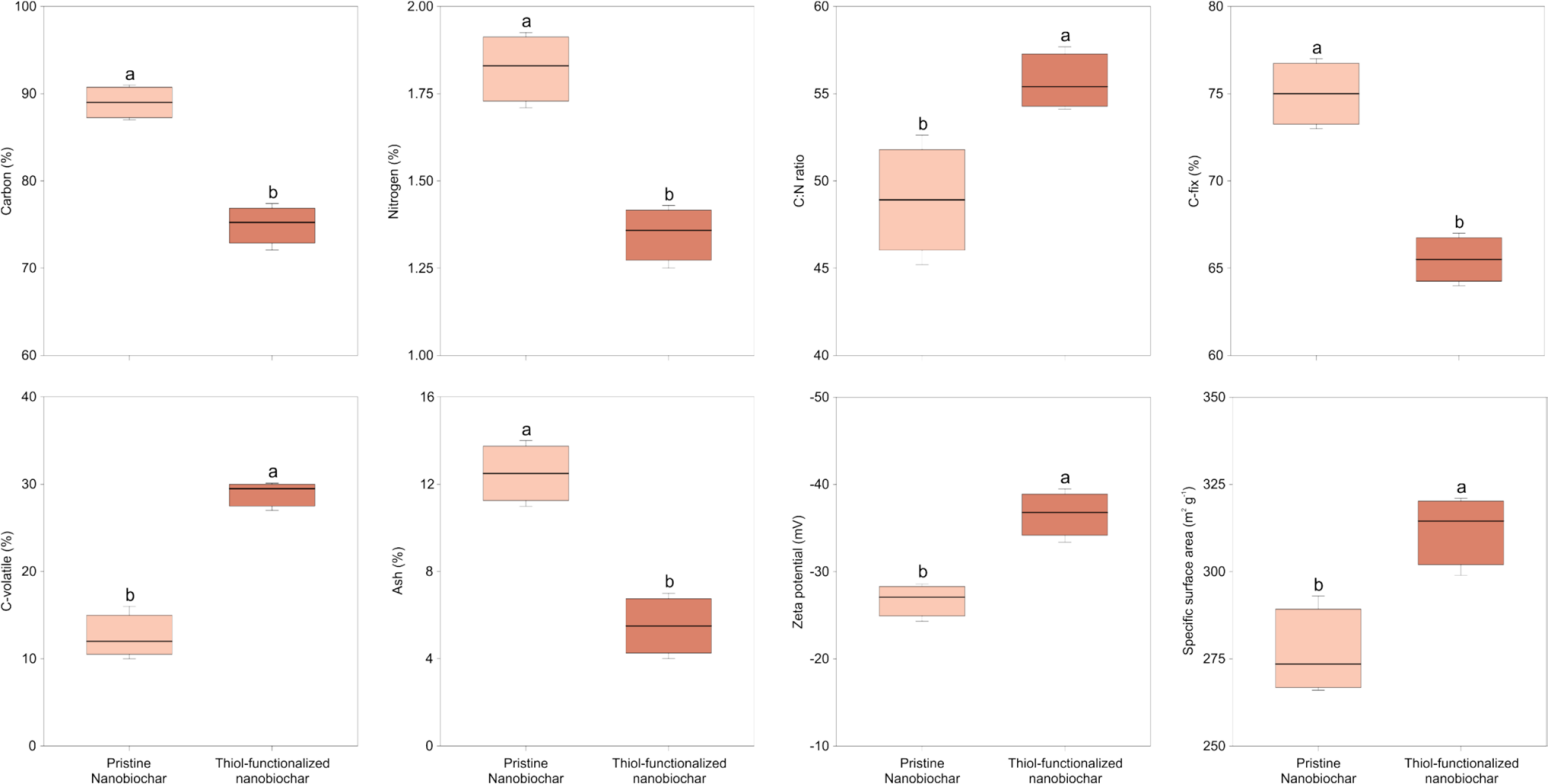
Box-and-whisker plots illustrating chemical and physical attributes of pristine and thiol-functionalized nanobiochar. Error bars are standard deviations (*n* = 3). Lowercase letters denote differences between treatments (Bootstrap test, *p* < 0.05)

Thiol (SH) functionalization significantly altered the physicochemical properties of nanobiochar (Fig. 3). Compared to pristine nanobiochar, the modified material exhibited lower contents of C, N, fixed C, and ash. These changes are likely related to the sorption of SH-containing compounds and the washing steps, which may have removed weakly bound organic matter and soluble ash components (Wei et al. 2022).

In contrast, the thiol-modified nanobiochar showed an increase in volatile carbon. This may be attributed to the presence of organic moieties introduced during functionalization, particularly from 3–MPTS, which can volatilize during proximate analysis.

Surface charge was also affected by SH functionalization. The ζ-potential increased by approximately 30%, indicating a higher density of negative charges on the surface (Fig. 3). This increase is associated with the incorporation of reduced sulfur groups (–SH), which contribute to surface charge and enhance electrostatic interactions (Lyu et al. 2020).

The specific surface area was also modified. Although numerical values suggest a decrease (from 312 to 276 m^2^ g^−1^), washing may have removed ash blocking micropores, potentially improving pore accessibility. During pyrolysis, ash can migrate and clog pores, particularly micropores, reducing surface area (Wang et al. 2022). The removal of these obstructions during functionalization may partially restore pore structure.

Chemical modification of biochar is widely recognized as an effective strategy to increase its specific surface area and to optimize its ability to interact with contaminants and its performance in environmental applications (Sizmur et al. 2017). Zhang et al. (2022), for example, demonstrated that activation with K_3_PO_4_ increased the surface area of ​​bamboo (*Bambusoideae*) biochar pyrolyzed at 550 °C from 156 to 275 m^2^ g^−1^. Liu et al. (2020) used ZnCl_2_ to modify biochar derived from Yunnan (*Pinus yunnanensis*) pine and observed an even higher increase, from 195 to 534 m^2^ g^−1^, under the same pyrolysis temperature. In comparison, the values ​​obtained in our study were lower, which can be explained by differences in the experimental conditions, such as the type of raw material and the chemical agent used and the pyrolysis parameters.

Chemical reagents play a key role in porosity development, with ZnCl_2_ promoting micropore formation and K_3_PO_4_ generating both micro- and mesopores depending on biomass composition. Pyrolysis conditions (e.g., temperature, heating rate, and residence time) also influence the final structure (Tomczyk et al. 2023). Lignin-rich biomasses, such as Yunnan pine, tend to respond better to chemical activation, yielding more porous materials. Thus, differences between values reported by Zhang et al. (2022) and Liu et al. (2020), and this study reflect variations in experimental conditions rather than limitations of the material.

**Fig. 4:**
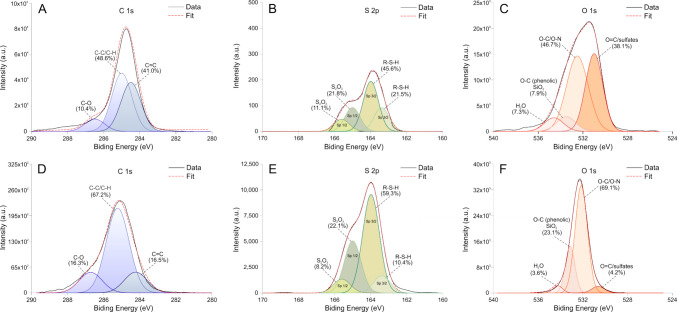
X-ray photoelectron spectroscopy analysis of carbon 1 s, sulfur 2p, and oxygen 1 s from pristine (**A**,** B**, and **C**) and thiol-modified (**D**, **E**, and **F**) nanobiochar

The addition of SH groups altered the surface chemical composition of nanobiochar (Fig. 4), particularly the distribution of carbon species (C 1 s). Pristine nanobiochar was dominated by C–C/C–H (48.6%) and C = C (41.0%) bonds, with a low fraction of oxygenated groups (10.4%). After SH modification, the proportion of C–C/C–H increased to 67.2%, while C = C decreased to 16.5%, indicating a higher density of aliphatic carbon. This shift likely reflects both a reduction in oxygenated groups, potentially bound to the silane group of 3–MPTS, and the contribution of aliphatic chains introduced during functionalization.

For S 2p, pristine nanobiochar contained both oxidized species (S = O, 21.8%; S–O₂, 11.1%) and reduced forms (R–S–H, 21.5%; R–S–R, 45.6%). After SH modification, the proportion of R–S–H increased to 59.3%, while oxidized sulfur fractions decreased (S = O to 22.1% and S–O_2_ to 8.2%). However, as these values represent relative proportions. The apparent decrease in oxidized species likely reflects an overall increase in total sulfur content rather than a reduction in their absolute concentrations.

For O 1 s, pristine nanobiochar was dominated by oxygenated groups, including O–C/O–N (46.7%) and O = C/sulfates (38.1%). After SH modification, O–C/O–N increased to 69.1%, while sulfates decreased to 4.2%, likely due to ash removal and improved surface accessibility. Overall, SH functionalization increased sulfhydryl groups and reduced oxidized oxygen and sulfur species (Fig. 4), enhancing specificity for Hg sorption due to its affinity for SH groups (Li et al. 2011) and indicating improved pore accessibility and reactive surface availability (Sarma et al. 2024).

The XPS spectra collected before and after the sorption of Hg^2+^ and CH_3_Hg^+^ revealed differences in the interaction and distribution of C, S, O and Hg elements between pristine and thiol-modified nanobiochar (Figs. A.1 and A.2). For C 1 s, a notable shift was observed after MeHg sorption, particularly in the proportion of O = C/SO_4_, which decreased from 38% in pristine nanobiochar to 5.5% after sorption (Fig. A.2C). This suggests that Hg binding altered the oxygenated functional groups and potentially affected the overall sorption mechanism.

Thiol-modified nanobiochar exhibited a greater proportion of C–C/C–H bonds after Hg sorption, with a concurrent reduction in C = O and C = C groups, indicating that the SH functionalization influenced carbon stability and Hg retention. Regarding S 2p, the proportion of R–SH groups remained high (up to 50.9%) after Hg^2+^ sorption, suggesting enhanced stability and a strong interaction with Hg species; a trend also observed for CH_3_Hg^+^. Conversely, pristine nanobiochar presented a greater variation in sulfur species, with a lower presence of R–S–H groups. So, SH functionalization not only increased the material’s selectivity but also made it more efficient in capturing Hg by stabilizing reactive sulfur groups, which are essential for the formation of compounds such as HgS (Rizwan et al. 2024).

For O 1 s, O–C/O–N groups were predominant in both materials, but thiol-modified nanobiochar had greater stability in interactions with O, with a higher proportion of these groups and a lower presence of sulfates (O = C/sulfates) after Hg^2+^ sorption. In contrast, after CH_3_Hg^+^ sorption, there was a decrease in the contribution of O–C/O–N and an increase in sulfates in thiol-modified nanobiochar, suggesting a different interaction mechanism for organic and inorganic Hg species. Even so, the behavior of thiol-modified nanobiochar reinforced its greater chemical stabilization capacity compared to pristine nanobiochar.

### Hg sorption performance of pristine and thiol-modified nanobiochars

**Fig. 5:**
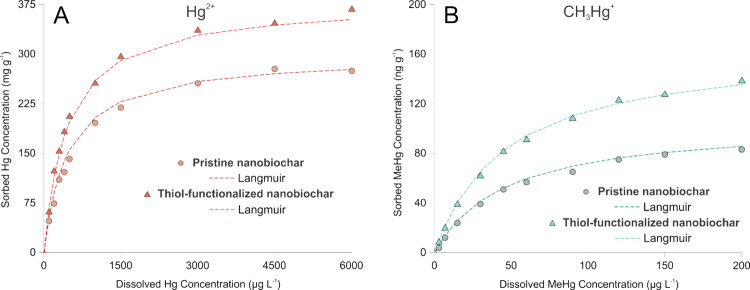
Mass-normalized sorption of mercury (**A**) and methylmercury (**B**) to pristine and thiol-functionalized nanobiochar at fixed conditions (pH = 7.8, 25 °C, and adsorbent dose of 1 g L^−1^). All data were modeled using the Langmuir sorption isotherm model, with fit parameters shown in Table A.1

Sorption experiments were conducted at fixed conditions (pH = 7.8, 25 °C, and 1 g L^−1^), representative of near-neutral environmental systems. Although parameters such as pH and temperature can influence Hg speciation and sorption behavior, the present study focuses on mechanistic insights rather than process optimization. Under these conditions, the sorption data for Hg^2+^ and CH_3_Hg^+^ (Fig. 5), along with the Langmuir isotherm fitting parameters (Table A.1), demonstrated that SH modification increased the maximum sorption capacity for both inorganic and organic Hg species. Pristine nanobiochar showed a Hg^2+^ maximum sorption capacity of 299 mg g^−1^, whereas thiol-modified nanobiochar reached 385 mg g^−1^, corresponding to an increase of approximately 29% (Fig. 5).

For CH_3_Hg^+^, pristine nanobiochar had a maximum sorption capacity of 102 mg g^−1^, and thiol-modified nanobiochar, of 164 mg g^−1^, showing an increase of approximately 61% due to SH modification. Despite the increase in the maximum sorption capacity (Table A.1), the *K*_*L*_ constant, which measures the affinity between the nanobiochar and the contaminant, remained similar between the two materials for both contaminants. This may suggest that SH enrichment impacts the amount of contaminant sorbed more than the initial interaction strength (Huang et al. 2023).

When compared to other studies, the nanobiochars used in our study had a higher capacity for Hg^2+^ retention. In pristine biochars, typical sorption capacities for Hg^2+^ range from 20 to 150 mg g^−1^, depending on the properties of the base material and the pyrolysis conditions (Mohan et al. 2014). Biochars derived from agricultural residues result in sorption capacities ranging from 13 to 135 mg g^−1^ (Rizwan et al. 2024). The 299 mg g^−1^ achieved using the pristine nanobiochar in our study exceeded these values, indicating the superiority of pristine nanobiochar. This greater maximum sorption capacity may be related to the properties, such as higher ζ potential and specific surface area, which generally improve after the transformation of biochar particles into nanoparticles.

Reducing particle size increases the surface area available for Hg sorption. Furthermore, modifying biochar into nanoparticles increases the number of functional groups, such as hydroxyl and carboxyl, that can bind Hg through ion exchange and complexation mechanisms. Transforming biochar into nanoparticles results in higher Hg removal efficiency than conventional biochar forms, due to these factors (Park et al. 2019; Zhang et al. 2024).

Functionalization with reduced S-containing groups, such as SH, is widely recognized to increase Hg^2+^ sorption due to the formation of Hg-S covalent bonds. In general, materials functionalized with reduced S have a Hg^2+^ sorption capacity between 17 and 624 mg g^−1^ (Wang et al. 2020), which makes the 385 mg g^−1^ maximum sorption capacity achieved using the thiol-modified nanobiochar in our study within the normal range expected for functionalized biochars. Therefore, the modification with SH positively affects the maximum sorption capacity because the functionalization introduces new high-energy sites that are relevant under conditions of high contamination (Wang et al. 2020).

Few researchers reported specific data regarding the retention of CH_3_Hg^+^. In general, biochars functionalized with sulfur groups have a CH_3_Hg^+^ sorption capacity between 80 and 180 ng g^−1^ (Wang et al. 2020; Yang et al. 2021). The maximum sorption capacity ​​of 102 ng g^−1^ for pristine nanobiochar and 164 ng g^−1^ for thiol-modified nanobiochar achieved in our study are within the range found in bibliographic references. When comparing pristine and thiol-modified nanobiochar, again, SH functionalization increase CH_3_Hg^+^ retention.

The greater CH_3_Hg^+^ sorption capacity after chemical modification of biochar may be associated with its retention in hydrophobic regions of the nanobiochar. Functionalization with -SH groups reduces the affinity of biochar for water and favors interactions between CH_3_Hg^+^ and organic domains of the material, enhancing retention (Hamid et al. 2022). Due to its partially hydrophobic character, CH_3_Hg^+^ can preferentially interact with carbon chains and aromatic structures on the functionalized surface, forming more stable associations and reducing its release (Huang et al. 2019). In addition, cation–π interactions between CH_3_Hg^+^ and aromatic rings may also contribute to the stabilization of these associations.

CH_3_Hg^+^ interacts preferentially with hydrophobic surfaces and sulfur-containing groups, which decreases its mobility and enhances its immobilization (Liu et al. 2016; Tanimu and Alhooshani 2021). In contrast, pristine nanobiochar exhibits a lower CH_3_Hg^+^ retention capacity due to the absence of chemical modifications that promote more specific and stable interactions with this species. Thus, the main mechanisms explaining Hg^2+^ and CH_3_Hg^+^ retention in thiol-modified nanobiochar include the formation of HgS, enabled by SH functionalization, and hydrophobic interactions in carbonaceous regions.

### Molecular mechanisms of Hg retention

**Fig. 6:**
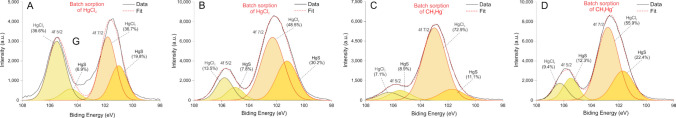
X-ray photoelectron spectroscopy analysis of mercury 4f on pristine (**A** and **C**) and thiol-modified nanobiochar (**B** and **D**) after batch sorption of mercury chloride (HgCl_2_) and methylmercury (CH_3_Hg^+^). The pristine nanobiochar retained 299 mg g^−1^ of Hg^2+^ and 102 ng g^−1^ of CH_3_Hg^+^, whereas the thiol-modified nanobiochar retained higher values (385 mg g^−1^ of Hg^2+^ and 164 ng g^−1^ of CH_3_Hg.^+^)

The Hg 4f spectra confirmed the superiority of thiol-modified nanobiochar for Hg immobilization (Fig. 6). A significant formation of HgS was observed, with 30.2% of Hg^2+^ being converted into HgS, corresponding to 116.3 mg of retained Hg^2+^, and 22.4% of CH_3_Hg^+^ undergoing the same transformation, equivalent to 36.7 ng of CH_3_Hg^+^. In contrast, pristine nanobiochar had a higher proportion of less stable species, such as HgCl_2_, which are more prone to remobilization under environmental conditions.

These findings demonstrate that thiol functionalization enhances the performance of nanobiochars, particularly by immobilizing more mobile and toxic forms of Hg, such as CH_3_Hg^+^. The observed retention, both in absolute amounts and in the stabilization of less stable species, underscores the potential of thiol-modified nanobiochar as an effective remediation strategy for Hg-contaminated environments.

The retention of Hg^2+^ and CH_3_Hg^+^ in nanobiochars can occur by mechanisms that vary according to the chemical modification of the biochar and the chemical form of Hg. Based on the XPS results (Fig. 6), the predominant Hg species identified on both pristine and thiol-modified nanobiochar surfaces are either HgCl_2_ or Hg bound to HS groups. In the case of pristine nanobiochar, the interactions primarily involve oxygenated functional groups, such as O–C/O–N, alongside associations with aromatic structures on the surface. However, despite Hg being predominantly identified as HgCl_2_ or HgS, the decrease in oxygenated functional groups, such as O–C/O–N, observed in the O 1 s spectra (Figs. A.1 and A.2) suggests that some Hg may still be bound to these sites on the nanobiochar.

XPS has limitations in detecting all Hg species, particularly those present in low concentrations or bound to less abundant functional groups. However, the ionic interaction mechanism presents limitations in the stabilization of Hg^2+^ and CH_3_Hg^+^, resulting in a lower efficiency for the conversion of Hg into more stable chemical forms, such as HgS. The direct binding of HgCl_2_ to O in biochar can inhibit the formation of more stable covalent bonds, such as HgS and HgO, by occupying reactive sites, which stabilizes Hg^2+^ in its less reactive HgCl_2_ form but may also limit its incorporation into more permanent immobilization phases. This interaction alters the reactivity of Hg, potentially compromising the effectiveness of Hg immobilization strategies in contaminated environments (Hsu-Kim et al. 2013; Liu et al. 2016).

In thiol-modified nanobiochar, SH functionalization introduces sulfur groups that increase the retention of Hg and CH_3_Hg^+^. The formation of HgS was identified as the main immobilization mechanism in this material, evidencing the high affinity between reduced sulfur groups (R–S–H) and mercurial species (Hg^2+^ and CH_3_Hg^+^). These results corroborate with other studies (Skyllberg et al. 2000; Gu et al. 2011), which indicate that reduced sulfur has a high complexation capacity with Hg, forming stable compounds, such as HgS.

**Fig. 7:**
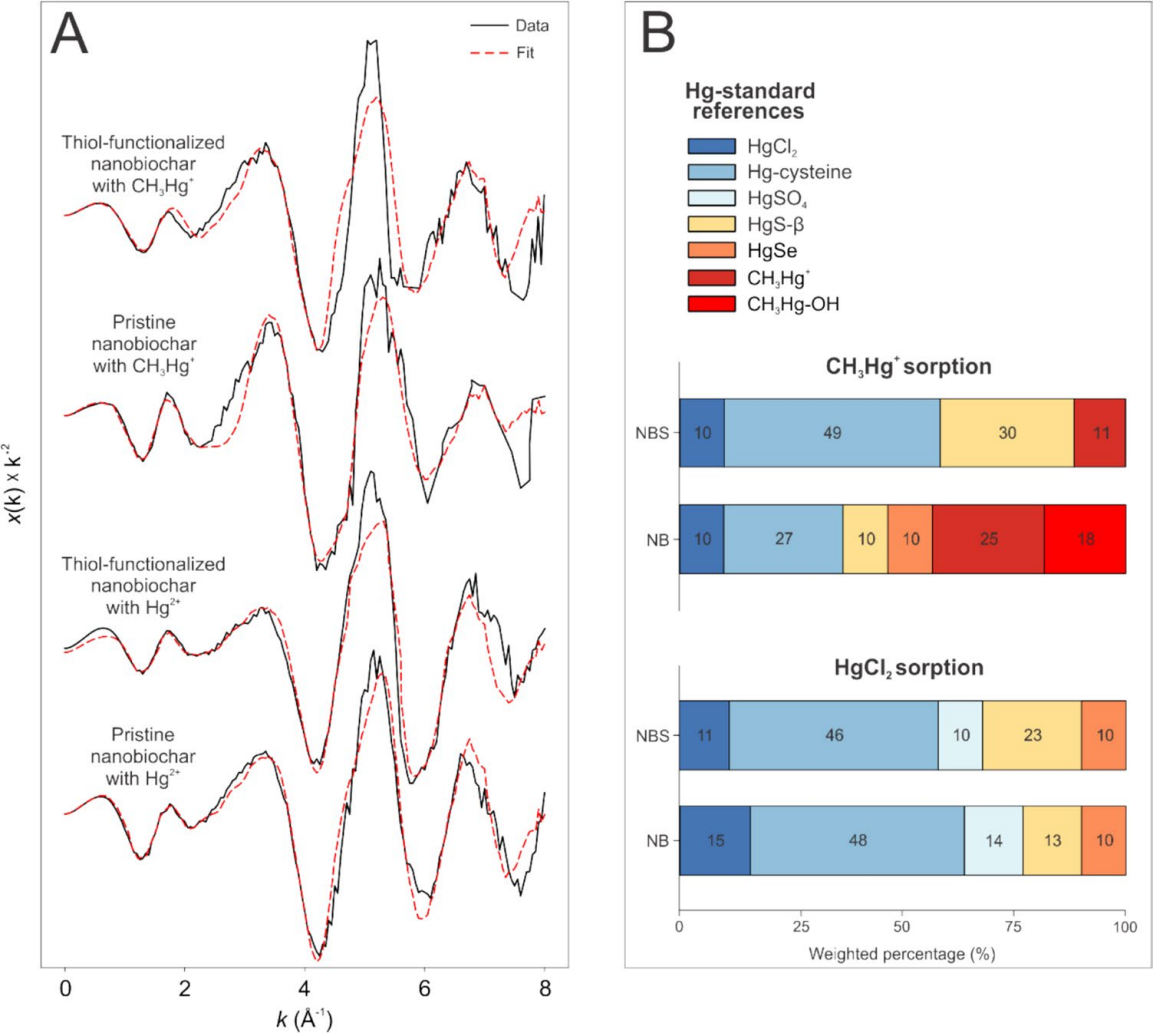
Hg L_3_-edge EXAFS spectra of pristine (NB) and thiol-modified (NBS) nanobiochar samples after sorption of HgCl_2_ (Hg^2+^) and methylmercury (CH_3_Hg^+^). **A** Experimental spectra (black line) and fits (red line). **B** Weighted proportions of Hg species determined by linear combination fitting (LCF). The pristine nanobiochar retained 299 mg g^−1^ Hg^2+^ and 102 ng g^−1^ CH_3_Hg^+^, while the thiol-modified nanobiochar retained 385 mg g^−1^ Hg^2+^ and 164 ng g^−1^ CH_3_Hg^+^

The Hg L_3_-edge EXAFS results confirmed the results obtained by XPS, in which Hg retention was higher with S compounds present in thiol-modified nanobiochar (Fig. 7). For the Hg^2+^ sorption, thiol-modified nanobiochar presented a higher proportion of Hg bound to S-containing groups (~ 79%), indicating that the sulfide in thiol-modified nanobiochar promoted the formation of more stable species, such as HgS–β. In the pristine nanobiochar, the contribution of Hg species associated with S was lower (~ 70%), while there is a greater presence of Hg bound to sulfates that can be retained in biochar, but with lower binding energy (Liu et al. 2016). The higher proportion of Hg binding to sulfur observed in EXAFS compared to XPS may be attributed to the greater probing depth of EXAFS, which provides information about bulk interactions within the material, while XPS is surface-sensitive and might underrepresent sulfur interactions occurring deeper in the matrix.

Thiol-modified nanobiochar resulted in greater retention of CH_3_Hg^+^ through Hg–S bonds, as found in HgS–β (Fig. 7B). However, in thiol-modified nanobiochar, no Hg species with direct bonds to carbon compounds (Hg–C) were identified in LCF (contributions above 10%), indicating that thiol-modified nanobiochar can inhibit the direct interaction of CH_3_Hg^+^ with organic compounds on its surface. On the other hand, in pristine nanobiochar there was a significant proportion of Hg–C (~ 18%), suggesting that pristine nanobiochar favored interactions of methylated Hg species with organic compounds present in its matrix, possibly through bonds with –OH functional groups. The identification of a greater proportion of Hg as HgS–β species highlights the potential of thiol-modified nanobiochar to be more effective in stabilizing Hg^2+^ and reducing CH_3_Hg^+^ mobility, possibly due to the formation of covalent bonds between Hg and S, in which Hg can be precipitated in forms similar to metacinnabar (HgS–β) (Mazrui et al. 2018; Wang et al. 2019).

A small proportion of the Hg present in the nanobiochar was identified in the form of HgCl_2_ (< 13%), which suggests that part of the retained Hg was associated with relatively weaker interactions, such as ionic bonds. This behavior is in line with bibliographic references, which show that sorbent materials, even when functionalized, can present residual fractions of Hg in less stable forms (Liu et al. 2016). Although the predominant retention of Hg in thiol-modified nanobiochar is highly effective, the presence of HgCl_2_ in both nanobiochars indicates a susceptibility to release under specific environmental conditions, such as changes in pH, ionic strength, or the presence of competitive ligands. This finding highlights a potential mobility risk that should be considered in the practical application of this material.

From an environmental point of view, these differences in the proportion of species formed in the nanobiochar may have important implications for the mechanisms of Hg retention in soils and water. In the case of thiol-modified nanobiochar, the mechanism was dominated by precipitation of Hg as sulfide (HgS) and possibly by complexation with thiol groups, which produce species with low bioavailability and high chemical stability. In pristine nanobiochar, the retention mechanism predominantly involved sorption on surfaces containing oxygenated groups, which may be less effective in stabilizing Hg under variable environmental conditions (e.g., changes in pH and Eh) (Bhandari et al. 2023).

### Hg desorption and stability of retained species

**Fig. 8:**
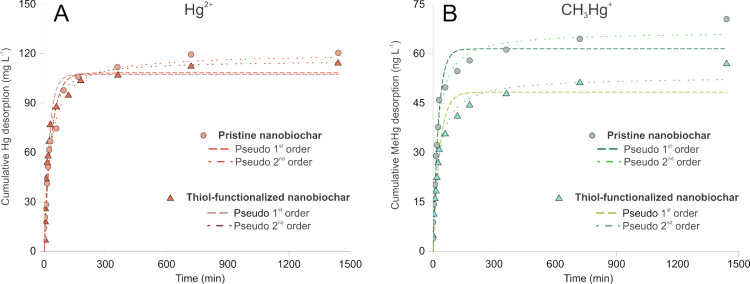
Cumulative mercury (**A**) and methylmercury (**B**) desorption from pristine and thiol-functionalized nanobiochar after extraction with 0.01 mol L^−1^ CaCl_2_ solution under conducted at controlled conditions (pH = 7.8, 25 °C, solid-to-solution ratio of 1 g L^−1^, and constant agitation), with cumulative release monitored over time

Thiol-modified nanobiochar had lower cumulative desorption for Hg^2+^ and CH_3_Hg^+^ compared to pristine nanobiochar, especially at the beginning of the experiment (Fig. 8). After approximately 600 min, desorption reached a plateau, indicating that the remaining Hg and CH_3_Hg^+^ were stable. This pattern demonstrates a rapid initial mobilization followed by a slower release over time, highlighting the stabilizing effect of thiol functionalization.

In the case of Hg^2+^, pristine nanobiochar exhibited a cumulative desorption of approximately 40%, while thiol-modified nanobiochar reached a lower value of around 30%. This finding is consistent with the observation that more of the Hg^2+^ and CH_3_Hg^+^ was in the HgS form in the thiol-modified nanobiochar (Figs. 6 and 7). The modification with SH groups improved the initial sorption of contaminants and stabilized these chemical interactions under ion exchange conditions (CaCl_2_). Kinetically, thiol-modified nanobiochar had a lower desorption rate, with a kinetic constant (k_1_​) of 0.02 g mg^−1^ min^−1^ in the pseudo-1st order model, compared to 0.04 mg^−1^ min^−1^for pristine nanobiochar. Furthermore, the equilibrium desorption capacity (*Q*_*e*_) was slightly higher for thiol-modified nanobiochar in both models, reaching 108 mg g^−1^ (pseudo-1st order) and 120 mg g^−1^ (pseudo-2nd order) (Table A.2). These results indicate that thiol functionalization enhances Hg retention and hinders its release.

For CH_3_Hg^+^, pristine nanobiochar exhibited a cumulative desorption of approximately 60%, while thiol-modified nanobiochar was closer to 45%. Regarding equilibrium desorption, pristine nanobiochar had a higher capacity, with *Q*_*e*_​ values of 61.3 ng g^−1^ in the pseudo-1st order model and 66.6 ng g^−1^ in the pseudo-2nd order model. Thiol-modified nanobiochar presented lower Qe ​values of 48.2 ng g^−1^ and 52.9 ng g^−1^ for the pseudo-1st and pseudo-2nd order models, respectively. This confirms that thiol functionalization reduced the mobility of CH_3_Hg^+^. The pseudo-2nd order model showed a better fit, reinforcing that the desorption process may involve specific chemical interactions between the contaminant and the adsorbent material.

The higher release of Hg^2+^ and CH_3_Hg^+^ from pristine nanobiochar is consistent with the Hg speciation identified by EXAFS (Fig. 7). In both pristine and thiol-modified materials, a fraction of Hg^2+^ occurs as HgCl_2_ (< 15%), which is associated with weaker ionic interactions and thus promotes desorption. This species may also derive from residual salts used during sorption experiments, potentially precipitated on the nanobiochar surface. Such behavior increases the risk of Hg release under environmental conditions that destabilize ionic bonds, including changes in pH, higher ionic strength, or the presence of competing ligands (Bollen et al. 2008).

In contrast, thiol-functionalized nanobiochar more effectively stabilized Hg^2+^ and CH_3_Hg^+^ through the formation of Hg–S covalent bonds (Fig. 7), which are more resistant to environmental perturbations, thereby reducing Hg mobility. Nevertheless, the presence of less stable species such as HgCl_2_ in both materials highlights the importance of considering environmental conditions when applying nanobiochar for Hg remediation.

When compared with other adsorbent materials, pristine and thiol-modified nanobiochar demonstrated superior retention of both Hg^2+^ and CH_3_Hg^+^. For Hg^2+^, thiol-modified nanobiochar retained 70%, while pristine nanobiochar retained 60%, exceeding the performance of biochar derived from rice (*Oryza sativa*) husks, which achieved Hg stabilization rates between 42 and 46% (Shen et al. 2021). However, biochars derived from switchgrass (*Panicum virgatum*) and poultry manure were more efficient, reducing Hg^2+^ concentrations in the solution by 80 to 90% (Liu et al. 2018).

For CH_3_Hg^+^, biochars derived from rice husks achieved removals between 36 and 72%, particularly when functionalized with SH groups (Xing et al. 2020). Biochars derived from switchgrass and poultry manure applied to sediments showed removals of 80 to 90%. Other biochars also demonstrated effectiveness, such as wheat (*Triticum* spp.) straw-derived biochar, which retained CH_3_Hg^+^ in the range of 55 to 71%, and bamboo (*Bambusoideae*) biochar, which achieved retention rates between 47 and 53% (Man et al. 2021). These findings emphasize that the type of biomass used as feedstock is a critical factor in determining the remediation efficiency of biochars across different environmental media, including soils, water, and sediments. Additionally, environmental parameters such as pH, ionic strength, and the presence of ligands significantly influence the performance of biochars and nanobiochars, underscoring the need for careful evaluation before practical application.

**Fig. 9:**
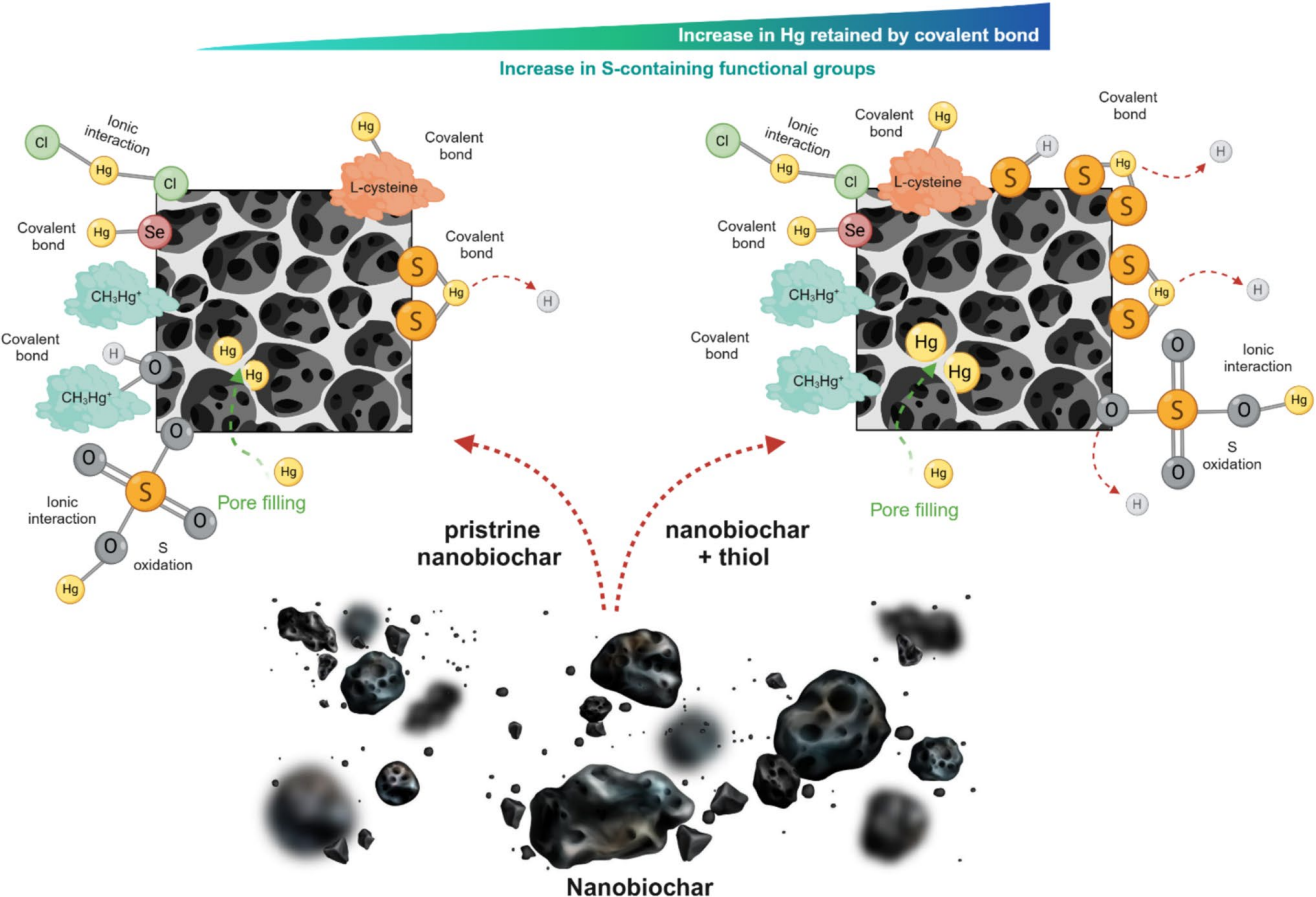
Mercury interaction model with pure and thiol-functionalized nanobiochar, based on XPS and EXAFS results, demonstrating the increase in Hg retention by covalent bonds mediated by sulfur-containing functional groups. The thiol-functionalized nanobiochar presented a higher density of sulfur-containing groups, increasing the Hg sorption capacity due to covalent bonds and ionic interactions. The results highlight the interaction with compounds such as L-cysteine, selenium, and methylmercury, evidencing the molecular mechanisms of mercury adsorption and complexation

The retention mechanisms of Hg^2+^ and CH_3_Hg^+^ in nanobiochar depend on the material's functionalization (Fig. 9). In pristine nanobiochar, sorption mainly occurs through ionic interactions and possible pore filling, which are less stable and easily disrupted by environmental changes, such as pH variation or competing ligands. This explains the higher desorption of Hg and CH_3_Hg^+^ observed. Conversely, thiol-modified nanobiochar forms covalent Hg–S bonds, which are more resistant to environmental fluctuations, enhancing contaminant retention and reducing desorption. These findings align with previous studies showing that thiol-functionalized biochars better stabilize mercury and methylmercury, lowering their mobility in soil and water. The lower desorption rates and stronger affinity of thiol-modified nanobiochar highlight its potential for remediating contaminated environments, particularly in sensitive areas like the Brazilian Amazon.

### Environmental application and practical limitations

The functionalization of nanobiochar with SH has proven to be a highly effective strategy for reducing the mobility of Hg^2+^ and CH_3_Hg^+^ species. Thiol-modified nanobiochar stands out for its lower desorption rate and greater chemical stability compared to conventional biochar and other adsorbent materials (Table A.3), making it an innovative solution for environmental remediation.

Thiol-modified nanobiochar has great potential for use as a filter material in water treatment systems contaminated with Hg, due to its high Hg adsorption capacity. This application is especially relevant in remote communities or regions with limited resources. Importantly, the thiol groups in this material are covalently bound to the nanobiochar surface and do not occur as free or volatile organic sulfur compounds. In addition, the material was thoroughly washed after functionalization to remove any unreacted residues. These factors minimize the risk of secondary contamination. However, further studies evaluating the potential release of organic compounds under real-world conditions are recommended to ensure environmental safety during large-scale applications.

From an economic perspective, biochar can be an advantage in remediation. For example, in 2020, biochar produced from wood and used in wastewater treatment was more cost effective (US$ 91–319/t), compared to activated carbon (US$ 1,500/t), zeolite (US$ 6,000/t) (Sun et al. 2022) and coconut shell-based granular activated carbon (US$ 3,500/t) (Made-in-China Co. 2026). However, the transformation into nanobiochar and its functionalization with SH may not be economically viable for widespread use as a soil amendment to remediate contaminated mine soils, especially in the northern region of Brazil.

Although nanobiochar has advantages in terms of efficiency and applicability, the costs associated with its production are substantially higher compared to conventional biochar. If we take into account the average cost of biochar production in Brazil (US$ 133–297/t) (Investbank 2024; Tomasi 2024), without considering the type of biomass or specific pyrolysis temperature, and add the average grinding cost in mining areas (US$ 58/t) (SOBRATEMA 2024) and the use of the 3-MPTS as SH source (US$ 730/t), the cost can be as high as US$ 921 to 1,085/t, making it less expensive than activated carbon, but unfeasible in remote areas of developing countries, such as Brazil. Therefore, the production of thiol-modified nanobiochar from biochar represents an increase in costs of 265–597% of the cost of the biochar. Furthermore, this cost could be much higher since related costs to washing the nanobiochar after using 3-MPTS were not calculated, and wastewater disposal was not considered in our calculations.

The high cost of thiol-functionalized nanobiochar for large-scale applications necessitates its use in high-value industrial settings where its unique properties offer substantial advantages. One promising application is in advanced water filtration systems, where its exceptional ability to retain Hg and CH^3^Hg^+^ through stable covalent bonding may enable compliance with stringent regulatory standards. This makes it a viable alternative to conventional filtration media in industries requiring high-performance contaminant removal.

Additionally, thiol-functionalized nanobiochar can replace activated carbon in industrial emission control systems, particularly in power plant chimneys, where it enhances mercury capture efficiency from combustion gases. Its superior adsorption capacity and chemical stability may contribute to reducing atmospheric Hg emissions and supporting environmental compliance efforts. Although the production costs of nanobiochar remain high, its targeted application in water and air purification systems justifies the investment due to its high contaminant retention efficiency and long-term environmental benefits.

## Conclusions

This study demonstrated the potential of nanobiochar, particularly thiol-functionalized nanobiochar, as an efficient material for the remediation of Hg^2+^ and CH_3_Hg⁺ in aqueous systems. The use of açaí seed residues, an abundant waste in the Brazilian Amazon, as a biochar feedstock highlights an approach aligned with circular economy principles and environmental sustainability.

Thiol functionalization of the nanobiochar significantly enhanced the sorption capacity and irreversibility of Hg and CH_3_Hg⁺ retention. This improvement is attributed to the introduction of reduced sulfur groups, which promote the formation of stable Hg–S covalent bonds, ensuring greater chemical stability under variable environmental conditions.

Thiol-functionalized nanobiochar shows strong potential for targeted applications, such as water treatment systems and emission control technologies, where stable Hg retention is required. Its performance suggests it could serve as an alternative to conventional materials like activated carbon, improving Hg capture efficiency in both environmental and industrial contexts.

Future research should focus on scaling up production, optimizing functionalization processes, and evaluating the long-term environmental stability of nanobiochar under realistic conditions. These efforts are essential to advance its practical application and support the development of effective strategies to mitigate Hg contamination globally.

## Supplementary Information

Below is the link to the electronic supplementary material.

ESM 1 (DOCX 761 KB)

## Data Availability

All the data of this study are contained in the manuscript. For any additional data or information required regarding this research, please contact the corresponding authors.
