# Supplementary material for: Mechanisms of mercury (Hg) and methylmercury (CH3Hg+) removal by pristine and thiol-modified açaí waste nanobiochar
Source: Environ Sci Pollut Res Int. 2026 Jul 21;33(24):12272–89. doi: 10.1007/s11356-026-38003-4 (PMC13468970; doi:10.1007/s11356-026-38003-4)
Supplement: Supplementary file 1 [file 11356_2026_38003_MOESM1_ESM.docx]

**Electronic Supplementary Information**

**Mechanisms of mercury (Hg) and methylmercury (CH_3_Hg^+^) removal by pristine and thiol-modified açaí waste nanobiochar**

*Matheus B. Soares^,1^, Luis C. C. Hurtarte^2,3^, Tom Sizmur^4^, Susan Nehzati^3^, Luís R.F. Alleoni^1^*

^1^ Department of Soil Science, Luiz de Queiroz College of Agriculture (ESALQ), University of São Paulo (USP), Piracicaba, 13418900, Brazil

^2^ Institute of Earth Surface Dynamics, University of Lausanne, Lausanne, 1015, Switzerland

^3^ Diamond Light Source, Harwell Science and Innovation Campus, Didcot, OX11 0DE, United Kingdom

^4^ Department of Geography and Environmental Science, University of Reading, Reading, RG6 6UR, United Kingdom

*(Contains 2 figures and 3 tables)*

**Table A.1.** Langmuir isotherm parameters obtained from the sorption results of mercury (Hg = Hg^2+^) and methylmercury (MeHg = CH_3_Hg^+^) on nanobiochar samples normalized to mass. Q_max_ = maximum sorption capacity; K_L_ = Langmuir's affinity constant; E = model efficiency on the scale 0 to 1.

| **Data set** | ***Q_max_* (mg g^-1^)** | ***K_L_* (L mg^-1^)** | **E** |
| --- | --- | --- | --- |
|  | ---------------------------------------------- Hg ----------------------------------------- | | |
| Pristine nanobiochar | 298.9 ± 2.7 | 0.021 ± 0.001 | 0.986 |
| Thiol-modified nanobiochar | 385.3 ± 1.9 | 0.025 ± 0.002 | 0.993 |
|  | ***Q_max_* (ng g^-1^)** | ***K_L_* (L ng^-1^)** | **E** |
|  | -------------------------------------------- MeHg --------------------------------------- | | |
| Pristine nanobiochar | 101.7 ± 0.7 | 0.024 ± 0.002 | 0.997 |
| Thiol-modified nanobiochar | 164.1 ± 1.2 | 0.023 ± 0.001 | 0.996 |

**Table A.2.** Kinetic parameters of mercury (Hg = Hg^2+^) and methylmercury (MeHg = CH_3_Hg^+^) desorption onto pristine nanobiochar and thiol-functionalized nanobiochar. Q_e_ = equilibrium desorption capacity; K = desorption constant; E = model efficiency on the scale 0 to 1.

| **Data set** | ***Pseudo 1^st^ order*** | | |  | ***Pseudo 2^nd^ order*** | | |
| --- | --- | --- | --- | --- | --- | --- | --- |
|  | *k_1_*  (g mg^-1^ min^-1^) | *Qe*  (mg g^-1^) | E |  | *k_2_*  (g mg^-1^ min^-1^) | *Qe*  (mg g^-1^) | E |
|  | ---------------------------------------------- Hg ----------------------------------------- | | | | | | |
| Pristine nanobiochar | 0.041 | 107.29 | 0.952 |  | 0.0004 | 115.91 | 0.983 |
| Thiol-modified nanobiochar | 0.028 | 108.28 | 0.928 |  | 0.0002 | 119.97 | 0.972 |
|  |  | | | | | | |
|  |  | | | | | | |
|  | *k_1_*  (g ng^-1^ min^-1^) | *Qe*  (ng g^-1^) | E |  | *k_2_*  (g ng^-1^ min^-1^) | *Qe*  (ng g^-1^) | E |
|  | -------------------------------------------- MeHg --------------------------------------- | | | | | | |
| Pristine nanobiochar | 0.039 | 61.33 | 0.958 |  | 0.0007 | 66.56 | 0.980 |
| Thiol-modified nanobiochar | 0.031 | 48.19 | 0.929 |  | 0.0007 | 52.89 | 0.967 |

**Fig. A.1.** X-ray photoelectron spectroscopy spectra of carbon 1s, sulfur 2p, and oxygen 1s of pristine (A, B, and C) and thiol-modified nanobiochar (D, E, and F) after batch sorption of HgCl_2_. Baseline subtraction was performed by the Shirley equation, and the peaks were deconvoluted by Gaussian-Lorentzian (Voigt function) equations. The full width at half maximum (FWHM) was allowed to fluctuate within 0.2 between the same element for pristine (NB) and thiol-modified nanobiochar (NBS). The pristine nanobiochar retained 299 mg g^-1^ of Hg^2+^, whereas the thiol-modified nanobiochar had a higher retention capacity (385 mg g^-1^ of Hg^2+^).

**Fig. A.2.** X-ray photoelectron spectroscopy spectra of carbon 1s, sulfur 2p, and oxygen 1s of pristine (A, B, and C) and thiol-modified nanobiochar (D, E, and F) after batch sorption of MeHg (CH_3_Hg^+^). Baseline subtraction was performed by the Shirley equation and the peaks were deconvoluted by Gaussian-Lorentzian (Voigt function) equations. The full width at half maximum (FWHM) was allowed to fluctuate within 0.2 between the same element for pristine (NB) and thiol-modified nanobiochar (NBS). The pristine nanobiochar retained 102 ng g^-1^ of MeHg, whereas the thiol-modified nanobiochar showed an enhanced retention capacity, retaining 164 ng g^-1^ of MeHg.

**Table A.3.** Adsorption capacities of (nano)materials for mercury removal from water and soil.

| Material | Size^1^ | Medium | Hg species | Adsorption capacity (mg g^-1^) | Reference |
| --- | --- | --- | --- | --- | --- |
|  | *Nanomaterials* | | | |  |
| Copper oxide | 20 nm | Water | Hg^2+^ | 5 ×10^−5^ | Raul et al. (2022) |
| Magnetic Fe_3_O_4_-Ag^0^ | 23–41 | Water | Hg^2+^ | 80%^ǂ^ | Iglezakis et al. (2020) |
| Silica mesopores with organic ligands of ammonium | 7.2 nm | Water | Hg^2+^ | 164 | Awual et al. (2016) |
| Amine and thiol groups in silica coated with Fe_3_O_4_ nanoparticles | 13.6 nm | Water | Hg^2+^ | 355 | Bao et al. (2017) |
| Silica mesopores doped with Thioglycolic acid | 2.35 nm | Water | Hg^2+^ | 43 | Kenawy et al. (2018) |
| L-cysteine functionalized  δ-FeOOH | 14 nm | Water | Hg^2+^ | 217 | Maia et al. (2019) |
| UiO-66-NH_2_ | 2.48 nm | Water | Hg^2+^ | 206 | Li et al. (2016) |
| Chalcogenide NP | N/A | Water | CH_3_Hg^+^ | 95%^ǂ^ | Wang et al. (2019) |
| Magnetic NP | N/A | Water | CH_3_Hg^+^ | 25 | Jiang et al. (2017) |
|  | *Carbon-based material* | | | | |
| Açaí seed-derived biochar | 30 nm | Water | Hg^2+^ | 299 | This study |
| 3-MPTS-modified açaí seed-derived biochar | 30 nm | Water | Hg^2+^ | 385 | This study |
| 3D spongin scaffold | N/A | Water | Hg^2+^ | 90%^ǂ^ | Domingues et al. (2021) |
| Nanoporous biochar-supported poly 2-aminothiophenol | N/A | Water | Hg^2+^ | 286 | Nejad and Sheibani (2022) |
| EDTA functionalized magnetic graphene oxide | 38 nm | Water | Hg^2+^ | 268 | Cui et al. (2015) |
| Raw corncobs biochar | N/A | Water | Hg^2+^ | 14 | Bilal et al. (2018) |
| Bagasse biochar | N/A | Water | Hg^2+^ | 13 | Xu et al. (2015) |
| Rice straw activated carbon | 120 μm | Soil | Hg^2+^ | 500 | Mashhadi et al. (2016) |
| Açaí seed-derived biochar | 30 nm | Water | CH_3_Hg^+^ | 1.02 ×10^−4^ | This study |
| 3-MPTS-modified açaí seed-derived biochar | 30 nm | Water | CH_3_Hg^+^ | 1.64 ×10^−4^ | This study |
| Rice hull biochar with thiol | N/A | Soil | CH_3_Hg^+^ | 49–73% | Xing et al. (2020) |
| Rice straw biochar | N/A | Soil | CH_3_Hg^+^ | 50% | Shu et al. (2016) |
| Sewage sludge biochar | N/A | Soil | CH_3_Hg^+^ | 73% | Zhang et al. (2019) |

^1^ N/A: information not provided by the author. ^ǂ^ The concentration was not provided by the authors, only the efficiency (%) was reported.

**References**

Awual MR, Hasan MM, Eldesoky GE, Khaleque MA, Rahman MM, Naushad M (2016) Facile mercury detection and removal from aqueous media involving ligand impregnated conjugate nanomaterials. Chemical Engineering Journal 290:243–251. https://doi.org/10.1016/J.CEJ.2016.01.038

Bao S, Li K, Ning P, Peng J, Jin X, Tang L (2017) Highly effective removal of mercury and lead ions from wastewater by mercaptoamine-functionalised silica-coated magnetic nano-adsorbents: Behaviours and mechanisms. Appl Surf Sci 393:457–466. https://doi.org/10.1016/J.APSUSC.2016.09.098

Bilal S, Fahim M, Firdous I, Ali Shah A-ul-H (2018) Insight into capacitive performance of polyaniline/graphene oxide composites with ecofriendly binder. Appl Surf Sci 435:91–101. https://doi.org/10.1016/J.APSUSC.2017.11.030

Cui L, Guo X, Wei Q, Wang Y, Gao L, Yan L, Yan T, Du B (2015) Removal of mercury and methylene blue from aqueous solution by xanthate functionalized magnetic graphene oxide: Sorption kinetic and uptake mechanism. J Colloid Interface Sci 439:112–120. https://doi.org/10.1016/J.JCIS.2014.10.019

Domingues EM, Gonçalves G, Henriques B, Pereira E, Marques PAAP (2021) High affinity of 3D spongin scaffold towards Hg(II) in real waters. J Hazard Mater 407:124807. https://doi.org/10.1016/J.JHAZMAT.2020.124807

Inglezakis VJ, Kurbanova A, Molkenova A, Zorpas AA, Atabaev TS (2020) Magnetic Fe3O4-Ag0 Nanocomposites for Effective Mercury Removal from Water. Sustainability 12:. https://doi.org/10.3390/SU12135489

Jiang W, Jin X, Yu X, Wu W, Xu L, Fu F (2017) Ion-imprinted magnetic nanoparticles for specific separation and concentration of ultra-trace methyl mercury from aqueous sample. J Chromatogr A 1496:167–173. https://doi.org/10.1016/J.CHROMA.2017.03.049

Kenawy IMM, Abou El-Reash YG, Hassanien MM, Alnagar NR, Mortada WI (2018) Use of microwave irradiation for modification of mesoporous silica nanoparticles by thioglycolic acid for removal of cadmium and mercury. Microporous and Mesoporous Materials 258:217–227. https://doi.org/10.1016/J.MICROMESO.2017.09.021

Li H, Zhu L, Wang J, Li L, Shih K (2016) Development of nano-sulfide sorbent for efficient removal of elemental mercury from coal combustion fuel gas. Environ Sci Technol 50:9551–9557. https://doi.org/10.1021/ACS.EST.6B02115

Maia LFO, Hott RC, Ladeira PCC, Batista BL, Andrade TG, Santos MS, Faria MCS, Oliveira LCA, Monteiro DS, Pereira MC, Rodrigues JL (2019) Simple synthesis and characterization of l-Cystine functionalized δ-FeOOH for highly efficient Hg(II) removal from contamined water and mining waste. Chemosphere 215:422–431. https://doi.org/10.1016/J.CHEMOSPHERE.2018.10.072

Mashhadi S, Sohrabi R, Javadian H, Ghasemi M, Tyagi I, Agarwal S, Gupta VK (2016) Rapid removal of Hg (II) from aqueous solution by rice straw activated carbon prepared by microwave-assisted H2SO4 activation: Kinetic, isotherm and thermodynamic studies. J Mol Liq 215:144–153. https://doi.org/10.1016/J.MOLLIQ.2015.12.040

Raul PK, Das B, Umlong IM, Devi RR, Tiwari G, Kamboj DV (2022) Toward a Feasible Solution for Removing Toxic Mercury and Chromium From Water Using Copper Oxide Nanoparticles. Frontiers in Nanotechnology 4:805698. https://doi.org/ 10.3389/fnano.2022.805698

Nejad MS, Sheibani H (2022) Super-efficient removal of arsenic and mercury ions from wastewater by nanoporous biochar-supported poly 2-aminothiophenol. J Environ Chem Eng 10:107363. https://doi.org/10.1016/J.JECE.2022.107363

Shu R, Wang Y, Zhong H (2016) Biochar amendment reduced methylmercury accumulation in rice plants. J Hazard Mater 313:1–8. https://doi.org/10.1016/J.JHAZMAT.2016.03.080

Wang X, Seelen EA, Mazrui NM, Kerns P, Suib SL, Zhao J, Mason RP (2019) The interaction of mercury and methylmercury with chalcogenide nanoparticles. Environmental Pollution 255:113346. https://doi.org/10.1016/J.ENVPOL.2019.113346

Xing Y, Wang J, Shaheen SM, Feng X, Chen Z, Zhang H, Rinklebe J (2020) Mitigation of mercury accumulation in rice using rice hull-derived biochar as soil amendment: A field investigation. J Hazard Mater 388:121747. https://doi.org/10.1016/j.jhazmat.2019.121747

Xu J, Bravo AG, Lagerkvist A, Bertilsson S, Sjöblom R, Kumpiene J (2015) Sources and remediation techniques for mercury contaminated soil. Environ Int 74:42–53. https://doi.org/10.1016/j.envint.2014.09.007

Zhang J, Wu S, Xu Z, Wang M, Man YB, Christie P, Liang P, Shan S, Wong MH (2019) The role of sewage sludge biochar in methylmercury formation and accumulation in rice. Chemosphere 218:527–533. <https://doi.org/10.1016/J.CHEMOSPHERE.2018.11.090>
